# The Cannabis Pathway to Non-Affective Psychosis may Reflect Less Neurobiological Vulnerability

**DOI:** 10.3389/fpsyt.2014.00159

**Published:** 2014-11-18

**Authors:** Else-Marie Løberg, Siri Helle, Merethe Nygård, Jan Øystein Berle, Rune A. Kroken, Erik Johnsen

**Affiliations:** ^1^Division of Psychiatry, Haukeland University Hospital, Bergen, Norway; ^2^Department of Clinical Psychology, University of Bergen, Bergen, Norway; ^3^Laboratory of Clinical Biochemistry, Haukeland University Hospital, Bergen, Norway; ^4^Department of Clinical Medicine, University of Bergen, Bergen, Norway

**Keywords:** cannabis, psychosis, schizophrenia, cognition, age of onset

## Abstract

There is a high prevalence of cannabis use reported in non-affective psychosis. Early prospective longitudinal studies conclude that cannabis use is a risk factor for psychosis, and neurochemical studies on cannabis have suggested potential mechanisms for this effect. Recent advances in the field of neuroscience and genetics may have important implications for our understanding of this relationship. Importantly, we need to better understand the vulnerability × cannabis interaction to shed light on the mediators of cannabis as a risk factor for psychosis. Thus, the present study reviews recent literature on several variables relevant for understanding the relationship between cannabis and psychosis, including age of onset, cognition, brain functioning, family history, genetics, and neurological soft signs (NSS) in non-affective psychosis. Compared with non-using non-affective psychosis, the present review shows that there seem to be fewer stable cognitive deficits in patients with cannabis use and psychosis, in addition to fewer NSS and possibly more normalized brain functioning, indicating less neurobiological vulnerability for psychosis. There are, however, some familiar and genetic vulnerabilities present in the cannabis psychosis group, which may influence the cannabis pathway to psychosis by increasing sensitivity to cannabis. Furthermore, an earlier age of onset suggests a different pathway to psychosis in the cannabis-using patients. Two alternative vulnerability models are presented to integrate these seemingly paradoxical findings

## Introduction

### Prevalence and clinical implications

Use of illicit drugs is common in non-affective psychosis (ICD-10 F20–29; (1)), usually seen in about half of the patients; 40–60%, ranging from 10 to 70% (2–9). Illicit drug use in psychosis has clinical implications and has been associated with more relapse and re-hospitalizations, poorer social functioning, medication non-adherence, heightened suicide risk, increased treatment needs, and worse clinical outcomes (10–17). However, there are few clinical differences in relation to symptoms and family loading between drug-using and non-drug-using patients (18).

KEY CONCEPT 1. Non-affective psychosis A broader diagnostic group than schizophrenia only; includes schizophrenia-spectrum psychosis, but not affective psychosis or drug-induced psychosis. The term reflects a more contemporary view of psychosis, e.g., as best reflected by a continuum.

Cannabis is the most widely used illicit drug in non-affective psychosis, and life-time cannabis use has typically been reported to be about 50% (2, 19–21). The rate of cannabis use disorder is somewhat lower; about every fourth patients with schizophrenia according to a recent meta-analysis by Koskinen et al. (22), with particularly high current and life-time rates in first-episode samples (28.6 and 44.4%, respectively). Cannabis is derived from the plant *Cannabis sativa*, and is usually used as an illicit substance in the form of dried flower buds (marijuana), resin from the trichomes (hashish), or various extracts collectively known as hashish oil. Cannabis has psychological and cognitive effects, and can have psychosis-imitating properties. These drug effects are usually attributed to cannabinoids, with delta-(9)-tetrahydrocannabinol (THC) as the main psychoactive substance influencing experience and cognition (23). There is large intra-individual variability in the psychological reactions to THC, possibly related to individual differences in the corresponding brain activation changes (24). Psychosis-prone individuals, people with psychosis, and others who are genetically vulnerable to psychosis have an increased sensitivity to the adverse effects of THC (23).

KEY CONCEPT 2. Cannabis The most widely used illicit drug worldwide; taken from the plant Cannabis sativa, and usually used as an illicit substance in the form of dried flower buds (marijuana), resin from the trichomes (hashish), or various extracts collectively known as hashish oil. Cannabis has psychological and cognitive effects, and can have psychosis-imitating properties.

KEY CONCEPT 3. THC Delta-(9)-tetrahydrocannabinol; a cannabinoid and the main psychoactive substance in cannabis, influencing experience and cognition.

### Cannabis use as a risk factor for non-affective psychosis

Longitudinal studies have reported an increased likelihood for developing schizophrenia and other psychoses after cannabis use (25, 26), especially when cannabis use has been moderate to severe and/or is started in the early teens (27–29). Schizotopy has also been associated with cannabis use in a recent meta-analysis (30). In addition, several large-scale longitudinal studies have reported a relationship between cannabis use in adolescence as well as later symptoms of sub-threshold psychosis in the general population (31–37). The relationship between cannabis and psychosis seems fairly specific to schizophrenia, as compared to other mental disorders (38–40), even though there are a relationship between symptoms of anxiety and cannabis (41). The relationship cannot be explained by potentially confounding factors such as premorbid disorders, other types of drug use, intoxication effects, personality traits, sociodemographic markers, or intellectual ability (40). Accordingly, several reviews conclude with an increased risk for psychosis in individuals who have used cannabis, typically in the magnitude of an odds ratio of 1.5–2 (20, 40, 42–44).

There are opposing views on cannabis as a risk factor for psychosis, however [see in Ref. (45) for an overview]. Some authors propose that there is a causal relationship between illicit drug use and non-affective psychosis (45, 46). Others suggest that illicit drugs only precipitate non-affective psychosis in vulnerable individuals on their pathway to psychosis (45–48). A variant of this is reversed causality; cannabis is used as a form of self-medication in psychosis, although existing data do not seem to support this hypothesis (38). Cannabis debut usually precedes onset of psychosis (10, 39, 49), e.g., by 7–8 years (50). However, most individuals do not develop psychosis after cannabis use, suggesting that risk of psychosis must be modulated by other factors. In line with this, data from recent comprehensive studies suggest that cannabis is an environmental risk factor interacting with more basic genetic and biological vulnerability for psychosis (51–53).

### The effect of cannabis on neurotransmitter systems

Tetrahydrocannabinol probably influences the endogenous cannabinoid and dopamine systems (23, 54, 55), via cannabinoid receptors, which are distributed with high density in the cerebral cortex, including brain regions implicated in schizophrenia, and influence dopamine synthesis and uptake (23). Most studies on the neurotoxicity of THC in general are based on animal models, suggesting that THC increases dopamine levels in several regions of the brain, including striatal and prefrontal areas (56). Animal studies have also found more irreversible residual effects in prepubertal rats after chronic exposure to THC as compared to more mature rats (57). THC is a cannabinoid receptor 1(CB1) agonist, and Casadio et al. (58) suggest that cannabis produces its effects via the influence on CB1 receptors on GABA and glutamate, which modulate the excitability of midbrain dopamine neurons and prefrontal cortical pyramidal cells; it thus appears to switch off inhibitory inputs to dopamine neurons. THC may aggravate dopaminergic imbalances by increasing the dopaminergic tone in striatal regions of the brain, which, when administered repeatedly, decreases dopamine levels in prefrontal regions of the brain via sensitization processes resulting in expressions of a psychotic disorder (56, 59). It is possible that the repeated administration of THC alters the functioning of the prefrontal cortex by acting on dopamine signaling via activation of CB1 receptors. Kuepper et al. (56) argue for interpretative caution, however, since most evidence is based on animal research and the effects of endocannabinoids are not yet fully understood. No relationship between striatal postsynaptic dopamine receptors and cannabis use has also been reported (60), and pretreatment with the dopamine receptor antagonist haloperidol did not alter the behavioral effects of delta-9-THC (23). Bloomfield et al. (61) on the other hand found that chronic cannabis use was associated with reduced presynaptic dopamine synthesis capacity in the striatum, suggesting a complex relationship between cannabis and changes of dopamine availability.

In addition, there may be psychoactive substances in cannabis not yet studied, as there are at least 85 different cannabinoids in cannabis (62). Cannabidiol (CBD) may have a buffer effect against the negative effects of THC, e.g., Schubart et al. (63) found an association between estimated CBD in cannabis and fewer web-based self-reported experiences of psychotic symptoms. The CBD/THC ratio is possibly of importance for the psychological effects of cannabis, suggesting that the increasingly popular THC-potent variants of cannabis (e.g., “skunk”) may be more psychosis-inducing. Finally, CBD has been under investigation in a few small clinical trials as a potentially novel antipsychotic agent, with equivocal results thus far and larger studies being needed (64). One promising study found CBD to be comparable in its antipsychotic effect to the antipsychotic amisulpride in a double-blind, randomized clinical trial in acute schizophrenia, and attributed the antipsychotic properties of CBD to its effect on the level of the endocannabinioid anandamide (65).

### Need for translational knowledge

Thus, there is a high prevalence of cannabis use in non-affective psychosis with clinical implications. Early prospective longitudinal studies conclude that cannabis use is a risk factor for psychosis, and biochemical studies on cannabis have suggested potential mechanisms for this effect. Recent advances in the field of neuroscience and genetics may have important implications for our understanding of this relationship. Importantly, we need to better understand the vulnerability × cannabis interaction to shed light on the mediators of cannabis as a risk factor for psychosis in order to create targeted interventions; e.g., for whom is cannabis precarious? Thus, the present study reviews recent literature on vulnerability variables and/or mediating variables and cannabis, including data from our own laboratory. This includes focus on age of onset, cognition, brain functioning, family history, genetics, and neurological soft signs (NSS) in non-affective psychosis. Models will be presented to explain the findings. This review is also based on previous work by the present authors in Frontiers in Psychiatry (66); putting the work into a broader context and outlining the implications for scientific questions and issues within the field.

## Age of Onset

Earlier psychosis debut – age of onset has been shown in patients who have been using cannabis (21, 67–71). However, some studies report no effect of illicit drug use on onset age (15, 72–74). The inconsistencies are possibly due to methodological differences and small sample sizes in several studies. Furthermore, some studies have used “first treatment contact” instead of first psychotic symptoms as an estimate for psychosis debut (69), which is sub-optimal since psychosis may last for several years before first treatment (75). Meta-analyses conclude, however, that there is an earlier onset age of psychosis in cannabis and polysubstance users compared with those without illicit drug use also when confounders have been controlled for (68, 76). In addition, there is an effect of THC dose – patients with a history of cannabis use had about 3 years earlier debut of psychosis, and the subjects who had been using high-potency cannabis everyday had the earliest onset (29).

KEY CONCEPT 4. Age of onset Age of psychosis debut, usually defined as the first psychotic breakthrough, defined by, e.g., a PANSS psychosis symptom item score of ≥4.

The earlier onset age can possibly be attributed to the illicit drug use acting on the developing brain in adolescence (29, 32, 77). This neurodevelopmental hypothesis is also supported by a stronger relationship between adolescent cannabis use and psychosis as compared to adult use (27, 28). The effect of cannabis timing is also supported by longitudinal studies. As compared to non-users, more adult psychosis was predicted by cannabis use at 15 versus 18 years (32) and cannabis use at around 15 years or younger versus older debut (78). Furthermore, Schubart et al. (79) found very early that both cannabis use (under 12 years of age) and heavy cannabis use (>25 €/week) were associated with an increased likelihood of psychiatric hospitalizations. A recent study of 410 first-episode psychosis patients reported that those who had started cannabis at age 15 years or younger had an earlier onset of psychosis for about 2 years, and that the users of high-potency cannabis everyday had the earliest psychosis onset (29). It is not possible to fully rule out, however, that the relationship between the development of psychosis and the younger age of cannabis debut is driven by a larger cumulative exposure to cannabis. Alternatively, early cannabis users may be the ones who have been using cannabis before the development of psychosis and thus cannabis could have been an actual risk factor for them. The older cannabis users, however, may have used cannabis more in parallel with the first signs of psychosis, hence clouding the dynamics of cannabis a risk factor. Still, transition to psychosis in an ultra-high-risk sample was found to be highest among those who started using cannabis before the age of 15 years and went on to frequent use (49).

The neurodevelopmental explanation is further substantiated by central brain development processes in adolescence that may be particularly sensitive to cannabis. Levels of endocannabinoids and cannabinoid receptors increase at this age, peaking at puberty (80). Furthermore, the endocannabinoid system is involved in key processes of brain maturation during adolescence, e.g., the control of neuronal specification and maturation (81). Thus, exposure to cannabis during critical neurodevelopmental stages may impact the maturation of the endocannabinoid system and other key neurotransmitter systems (58). Bossong and Niesink (55) suggest that cannabis use during adolescence results in the disturbance of certain local neural circuits within the prefrontal cortex, and that the disturbance occurs as an interaction between THC and the CB1 receptors involved in the control of GABA and glutamate release. In line with this, studies have found that age at first use of cannabis predicted age at first psychotic symptom in patients with recent non-affective psychosis (50, 77, 82).

Our laboratory in Bergen, Norway, together with collaborating study organizations in Oslo and Stavanger, examined the effect of illicit drug use on onset age in a large sample of 1,119 patients with non-affective psychosis (83). Patients with illicit drug use had a significantly lower onset age of about 3 years compared with the abstinent group, primarily related to cannabis use and not to alcohol or other substances. This supports the notion that the effect of cannabis on age of onset is specific. As an overall conclusion, there is now evidence for an earlier onset age of psychosis in cannabis users often reported to be about 2–4 years. There are few studies suggesting that the earlier onset is related to early initiation of cannabis use, e.g., before the age of 15 years and related to neurodevelopmental processes, but there may also be alternative explanations for this relationship.

## Cognition

A majority of patients with schizophrenia and non-affective psychoses have clinically significant cognitive deficits (84–87). Cognitive deficits are vulnerability markers, present before the development of psychosis (87, 88), in high-risk populations (89, 90), and which persist after treatment of clinical symptoms (91, 92). General cognitive dysfunctions across cognitive domains are present, with additional selective deficits in working memory, executive function, attention, verbal fluency, episodic memory, and processing speed (93, 94). Typically, the impairment is between 1 and 2 SD, indicating a clinically significant loss of function (87, 95–98). Cognitive functioning is more important than positive psychotic symptoms in determining the patient’s functional outcome (93, 99–102).

KEY CONCEPT 5. Cognitive deficits Reduced ability or capacity for mental information processing; in non-affective psychosis often seen as problems in the following areas: attention/vigilance, working memory, learning, executive functioning, perceptual processing, visuomotor speed, and verbal fluency.

There have been difficulties in drawing firm conclusions regarding the long-term effects of cannabis on cognition, and studies should be interpreted with caution, however, due to often uncertain abstinence periods (103). Decrements in the ability to learn and remember new information in chronic users have been suggested, whereas other cognitive abilities are more unaffected, and younger cannabis users may be particularly vulnerable to these effects (104, 105). This age affect is, however, not substantiated by many studies. Studies making a distinction between adolescence and adult debut have shown some cognitive decrements in early-onset cannabis users (106, 107).

Cannabis use may affect cognition differentially in psychosis. In a preliminary study in our laboratory, we examined the effects of cannabis on cognition in 31 patients with schizophrenia (19, 108). Surprisingly, we found that patients with schizophrenia who had a history of cannabis use scored significantly better than psychosis patients without cannabis use. This was found for almost all cognitive domains investigated except learning; general intellectual ability, executive functions, attention, working memory, and psychomotor speed. These results did not change when other illegal drugs where controlled for, and there were no differences in the two groups with regard to clinical variables (19). We hypothesized that there was less cognitive vulnerability in the cannabis group. Interestingly, other authors, such as Schnell et al. (109), also suggested similar explanations at this point in time.

This finding prompted a review of the existing literature on the relationship between cannabis use and cognitive functioning in non-affective psychosis, and a systematic PubMed search resulted in 23 studies (110). Fourteen of the studies reported that the cannabis groups showed better cognitive performance than the no-cannabis groups (19, 73, 108, 109, 111–120). Eight of the studies reported no or minimal differences in cognitive performance in the two groups (21, 121–127), and one study reported better cognitive performance in the no-cannabis compared with the drug group (128).

The results showed that most studies had found better cognitive functioning in psychosis patients with cannabis use compared with psychosis alone (110). This pattern has been found by other meta-analysis (112, 119, 129) and replicated by more recent studies (130, 131) as well as two meta-analyses (132, 133). There are also studies with contradictory findings, possibly due to methodological issues such as different definitions of drug abuse (110); there are methodological differences related to ongoing versus previous drug use, frequent versus infrequent use, younger age in drug groups, and differences in relation to test batteries and cognitive domains examined. However, superior cognitive functioning in the drug-using group has been reported in first-episode psychosis patients (131, 134) and at 10-year follow-ups after onset of psychosis (116). The meta-analysis by Yücel et al. (132) found that cannabis-using patients performed moderately better than non-using patients on measures of global cognition, visual memory, processing speed, working memory, planning, and reasoning. Rabin et al. (133) reported the following effect sizes for superior cognitive functioning in cannabis-using patient compared with non-using patients for each neurocognitive domains in their meta-analysis: general cognitive ability and intelligence 0.48; selective, sustained and divided attention 0.35; executive abilities 0.14; working memory and learning 0.07; retrieval and recognition 0.12; receptive and expressive language abilities 0.06; and visuospatial and constructional abilities 0.33; these thus show effect sizes in the small to moderate range.

To explain the different cognitive profiles, we hypothesized that there was less cognitive vulnerability in the cannabis group; their psychosis breakthrough was related to aberrant information processing due to cannabis-induced transient cognitive deficits. To test this, we first performed a preliminary prospective study of 31 patients with acute psychosis, assessing cognitive function at admission to a psychiatric emergency ward, after 6 weeks and after 3 months. The patients with both cannabis history and psychosis showed a significantly larger improvement in their cognitive performance in the 3 months after admission compared with the psychotic patients without cannabis use (108).

In a continuation of this study, we improved the design by focusing on the period when the patients were in-patients to control for the illicit drug use after admission to the acute ward in a total sample of 123 patients (135, 136). The patients were examined by the Battery for the Assessment of Neuropsychological Status (RBANS) with alternative forms to minimize practice effects (95, 137, 138) at baseline and follow-up (mean time to follow-up 4 weeks). As expected, the cannabis-using group showed the largest improvement in cognition, especially among the youngest patients. This suggests that indeed cannabis use did induce transient cognitive deficits in the cannabis-using psychosis group, and that younger patients could have a larger capacity for restoring their cognitive capability. Thus, overall, there seems to be less persistent cognitive deficits in patients with psychosis and cannabis use, indicating less neurocognitive vulnerability.

## Brain Functioning

Reviews of structural brain imaging studies in non-affective psychosis, usually by means of magnetic resonance imaging (MRI) paradigms, have shown several distinct alterations in gray matter and white matter, both widespread and, in some studies, progressive changes (139). Voxel-based-morphometry (VBM) has, according to a review, most consistently shown volume reduction in the superior temporal cortex in chronic patients, and in frontal brain regions in first-episode and high-risk individuals (140). Functional brain imaging studies, often by means of functional MRI (fMRI), find abnormal communication between and/or integration of brain activation in local and distributed circuits (141–143), and connectivity deficits and additional transient states of hyper- and/or hypo-connectivity related to specific tasks (142). Decreased brain activation to effort-demanding tasks is a typical finding in non-affective psychosis (144–147), and lately several studies have shown increased brain activation in the default mode network (which includes activation in the medial prefrontal and temporal lobes reflecting endogenous generated thought) in psychosis compared with normal controls (147–150).

KEY CONCEPT 6. Brain imaging Techniques to image the structure and function of the brain, typically by means of magnetic resonance imaging (MRI) and functional magnetic resonance imaging (fMRI). There are also other brain-imaging techniques.

Studies have mostly been inconclusive in regard to the effect of long-term exposure to cannabis on the brain, but some brain imaging studies have found effects of long-term heavy use on gray matter volume (151–154). Mata et al. (155) suggested that normal neurodevelopment was affected after observing gyrification abnormalities in the cortex after long-term use. Long-term cannabis use has also been found to affect white matter as measured by diffusion tensor imaging (DTl), e.g., in the corpus callosum (156). Bossong et al. (157) recently reviewed neuroimaging studies on the long-term and acute effects of cannabis in both adult and adolescents on brain function related to learning and memory functions. Cannabis did not affect performance, but there were subtle changes in brain activation patterns in the cannabis-using group – both acute and long-term effects. The authors concluded that there was increased activity and a higher level of deactivation in the cannabis groups, and attributed this to compensatory increased or changed neural effort or non-cognitive factors like cerebral perfusion; however, they also pointed to the methodological problems related to comparing the studies.

Structural MRI and DTI studies comparing psychoses with and without cannabis use have shown less altered (134, 158), more anomalous (159–164), and equivalent (165–167) brain anatomy in the cannabis group, thus making firm conclusions difficult. Ongoing, long-term, and heavy use may influence the results: the continuation of cannabis use was found to increase gray matter loss and lateral and third ventricle enlargement after 5 years (161), and users of more than five joints daily for more than 10 years were shown to have bilaterally reduced hippocampal and amygdala volumes (168). A systematic review of 15 MRI and 4 post-mortem studies found evidence for brain structural abnormalities after cannabis use in psychosis in CB1-rich areas of the brain-like cingulum, the dorsolateral prefrontal cortex, and the cerebellum, and the authors suggested that the effect of cannabis is actually more distinct in psychosis than in the normal controls (154). Smith et al. (169) compared healthy controls, subjects with a history of cannabis use disorder, schizophrenia only, and both schizophrenia and a history of cannabis use disorder, while subjects with recent cannabis use were excluded. Both cannabis groups showed differences in relation to WM-related subcortical morphology, and the authors attributed this to either chronic cannabis abuse or the presence of biomarkers that characterize a vulnerability to the effects of cannabis.

There have been too few fMRI studies on long-term cannabis use and non-affective psychosis to reach a conclusion. One study, however, reported that schizophrenia patients with substance-use history showed increased cerebral activation to passive viewing of emotionally negative pictures, and concluded that the medial prefrontal cortex functioning is more preserved in dual-diagnosis schizophrenia (170). Potvin et al. (171) also found less impaired brain functioning during socio-emotional processing in patients with a dual diagnosis (mainly cannabis users) than schizophrenia alone.

To further examine brain functioning in cannabis use and psychosis, we conducted a study at our laboratory (66) to examine brain activation in 26 patients with schizophrenia with and without a history of previous cannabis use by using an fMRI paradigm comparing task-dependent (effort mode network) [cf. Ref. (172)] and task-independent (default mode network) [cf. Ref. (173)] conditions. We expected to replicate the better cognition for the cannabis users by finding less-anomalous brain activation patterns in the previous cannabis group, defined as the ability to up-regulate the effort mode network during the task-dependent condition [dichotic listening task with attention instructions, see in Ref. (174)] and down-regulate the default mode network during the task-independent condition. The present sample did all have a history of cannabis abuse as the main drug of choice, but not within the last 6 months, and patients that had been using meth-amphetamine, cocaine or opiates were excluded. The study showed different activation patterns for the task-dependent and task-independent conditions, essentially following the effort and default mode networks, respectively. Although all patients showed similarities across activation patterns, group differences emerged in the intensity and extension of the activation patterns that could not be explained by differences in clinical or demographic variables. The activation was more pronounced for the task-dependent condition in the cannabis group, while it was more pronounced for the task-independent condition in the no-cannabis group. Thus, as hypothesized, the previous cannabis group managed to up-regulate the effort mode network during the task-dependent condition and down-regulate the default mode network during the task-independent condition to a larger extent than the no-cannabis group. The no-cannabis group did show a pattern closer to the typical schizophrenia findings [see in Ref. (147)], indicating more impaired brain activation, and supporting less-anomalous brain activation in cannabis psychosis (66).

Two later studies have similar findings of better brain functioning for cannabis users. Patients with dual diagnosis of cannabis abuse and schizophrenia were found to be less impaired relative to schizophrenia only compared with healthy controls in regard to emotional memory and prefrontal lobe functioning (175), and showed a more normalized brain activation pattern during mental rotation in the left superior parietal region relative to schizophrenia only compared with healthy controls (176). It must be noted, however, that the differences between the different groups are subtle in most studies, and even group-based differences between normal controls and patients with schizophrenia and non-affective psychosis are usually subtle and of unclear clinical relevance. Still, it can be concluded that there are some data, although scarce, suggesting minor long-term effects of cannabis on brain structure in psychosis patients, but that the majority of studies show better brain functioning in this group, suggesting less neurocognitive vulnerability.

## Other Variables Mediating Neurobiological Vulnerability

### Neurological soft signs

Neurological soft signs are subtle sensory and motor performance anomalies that serve as markers of sub-optimal neurological development – there is an excess of NSS already evident in first-episode psychosis (177). Fewer NSS have been observed in schizophrenia with cannabis use than without cannabis use (74, 116, 160, 178). Ruiz-Veguilla et al. (178) reported an association between high NSS and not having been a heavy cannabis user and a family history of psychosis, and suggested a potentially different pathway to psychosis in relation to cannabis use. Less NSS in cannabis psychosis indicates less neurobiological vulnerability.

KEY CONCEPT 7. Neurological soft signs Subtle sensory and motor performance anomalies that serve as markers of a sub-optimal neurological development.

### Family history and genetics

Family history of serious mental disorders is often used as a proxy for genetic vulnerability (179). Proal et al. (180) reported that those who developed schizophrenia after cannabis use in adolescence had the same family history of schizophrenia as patients without cannabis use.

A few studies have examined the effects of polymorphisms in candidate genes on cannabis use and psychosis. In a longitudinal study, an interaction between the valine versus methionine allele of the Catechol-*o*-methyltransferase (COMT) gene and adolescent cannabis use significantly increased the likelihood of psychosis (27). This finding has not been replicated by all studies (179), however; Zammit et al. (181) did not find an effect of cannabis use on psychosis according to variation in COMT alleles, but an interaction between the COMT alleles and sensitivity for psychosis and cognitive effects of THC has been replicated by Henquet et al. (182, 183). There are also other genes, influencing D2 receptors and the cannabinoid system, that have been found to modify the sensitivity to cannabis. Certain variant (C/C genotype) of the AKT1 gene has been shown to give an increased likelihood of psychosis after life-time cannabis use (29), and looking at schizotypy proneness in unaffected siblings, Van Winkel and Genetic Risk and Outcome of Psychosis (GROUP) Investigators (52) found that genetic variation in AKT1 mediated both short-term as well as longer-term effects associated with use of cannabis.

This indicates a gene × environment interaction (179); there is possibly a genetically based sensitivity to substances (59), consistent with the fact that most people do not develop psychosis after cannabis use. Parakh and Basu (184) reviewed evidence on the association between cannabis and psychosis and concluded that cannabis is a component cause of psychosis, increasing the risk in people with certain genetic or environment vulnerabilities. Verdoux (185) found that subjects with established vulnerability for psychoses showed a stronger risk for psychosis after cannabis use than individuals without such vulnerability. Still, a study observed that cannabis also increased the risk for schizophrenia when family history was controlled for (78). In addition, cannabis interacts with other environmental risk factors like developmental trauma and child maltreatment, minority group position, and growing up in an urban environment, increasing the risk even further with the increasing number of risk factors (51, 59, 186). Also, certain variants of the COMT gene gave an increased risk for psychotic experiences after exposure to both cannabis use and childhood maltreatment (187, 188). It has been suggested that the environmental risk factor may induce differential sensitivity to cannabis (59). Recent animal models suggest long-lasting epigenetic changes after early negative life events (189), thus epigenetic mechanisms may increase the vulnerability to cannabis in certain individuals.

KEY CONCEPT 8. Gene × environment interaction The interaction between genetic vulnerability and environmental risk factors for psychosis, exemplified by the notion of a genetically based sensitivity to illicit substances such as cannabis.

Summing up, there seems to be some familiar and genetic vulnerability present in non-affective psychosis with cannabis use, possibly modified by environmental risk factors, that may be acting on neurotransmitter systems and/or genetic expression that particularly sensitize the individual to cannabis, but there are also less vulnerability markers such as NSS, cognitive deficits and probably brain abnormalities than in psychosis without cannabis use.

## Discussion

### Critical remarks

There are inconsistencies – not all studies find better cognition or less-disturbed brain functioning in cannabis-using patients. An important variability in the methodology of the studies may explain this, e.g., the different definitions of cannabis use, current, life-time or previous use, or a cannabis use disorder. Previous use, before the development of psychosis, is most relevant for the effects of cannabis within a vulnerability framework (110). It is also important to rule out the potential confounding effects of cannabis intoxication or recent cannabis use on, e.g., brain functioning seen in some studies (190–192). Recently, a large cross-sectional study compared the effect of current cannabis use and the effect of life-time cannabis use in 956 patients. This study concluded that there was a short-term negative effect of recent cannabis use on cognition and in contrast a positive long-term effect of life-time use, suggesting that the life-time cannabis-using group formed a subgroup with a different cognitive profile (193). Furthermore, some studies exclude patients not meeting the diagnostic criteria of a substance-use disorder, biasing the drug groups to consist of quite heavy users, and there are even studies where the no-drug group includes patients with previous drug use [see Ref. (122)].

Although cannabis as a risk factor for psychosis is quite established, there are unresolved issues. Epidemiologically, it is hard to explain that there is no great increase in the prevalence of non-affective psychosis in light of the increasing use of cannabis in several countries, even though a few authors argue that this is indeed the case (194). Furthermore, most patients do start using cannabis before psychosis breakthrough (73, 195–197). But developmental processes related to the disorder itself can theoretically influence the inclination to take drugs, and this is hard to test empirically.

Theoretically, there may be alternative explanations for the better brain and cognitive functioning in cannabis psychosis, e.g., superior social skills among those with cannabis psychosis, making users “skillful” enough to get hold of illegal drugs. Superior social skills are not consistent with the finding of poorer prognosis in this group. To our knowledge, few longitudinal studies have examined this directly and the issue remains unresolved. Two studies reported poorer premorbid functioning in psychosis patients who also used illegal drugs (198), and better premorbid social functioning and poorer premorbid academic functioning in this group (199), respectively.

Understanding the path to psychosis via cannabis also needs to take into account complex psychological and motivational processes. Griffith-Lendering et al. (200) assessed self-reported thought problems, social problems, attention problems, and cannabis use at different time points during adolescence. Cannabis use predicted psychosis vulnerability and vice versa, suggesting a bidirectional relationship between these variables, even though the concept of self-medicating is misleading due to cannabis’ negative influence on symptomatology (201). Using a time-sampling technique, daily life cannabis use was shown to predict a more acute decrease in negative affect but also sub-acute increased levels of hallucinatory experiences in patients than controls, suggesting a vicious circle of deleterious use in these patients (201). In-depth interviews showed that the patients themselves reported change over time in their experience of cannabis, cannabis use preceded mental health for all; cannabis use was fun at first, but the experience changed over time to more addiction-based behavior and more frightening experiences, but also parallel positive experiences for some (202).

### Vulnerability models to explain the findings

Taken together, there are now findings from studies on age of onset, cognition, brain functioning, NSS, and family history that together make a pattern. Compared with non-using non-affective psychosis, the present review shows that there seems to be less stable cognitive deficits in patients with cannabis use and psychosis, in addition to less NSS and possibly more normalized brain functioning, indicating less neurobiological vulnerability for psychosis. There is, however, some familiar and genetic vulnerability for psychosis present in the cannabis psychosis group. The familiar and genetic vulnerability may possibly influence the cannabis pathway to psychosis by acting on the neurotransmitter systems that may be particularly sensitive to cannabis, with several recent comprehensive literature reviews supporting this conclusion (45, 51, 55, 56, 58, 179, 184, 186, 203).

This gene–environment interaction does not, however, integrate the seemingly paradoxical findings of both less vulnerability markers and some genetic vulnerability. Bloomfield et al. (61) also suggested that cannabis increased the risk of psychosis by a different mechanism than typically seen in schizophrenia after examining dopamine synthesis capacity in the striatum. Furthermore, an earlier age of onset suggests a different pathway to psychosis in cannabis-using patients. It can be speculated that there are different levels of vulnerability in cannabis psychosis and no-cannabis psychosis, both groups having more general genetic vulnerability, while the cannabis pathway to psychosis has less neurobiological vulnerability. This can reflect two alternative models; (1) less specific vulnerability factors for psychosis, or (2) less general vulnerability.

**Figure 1:**
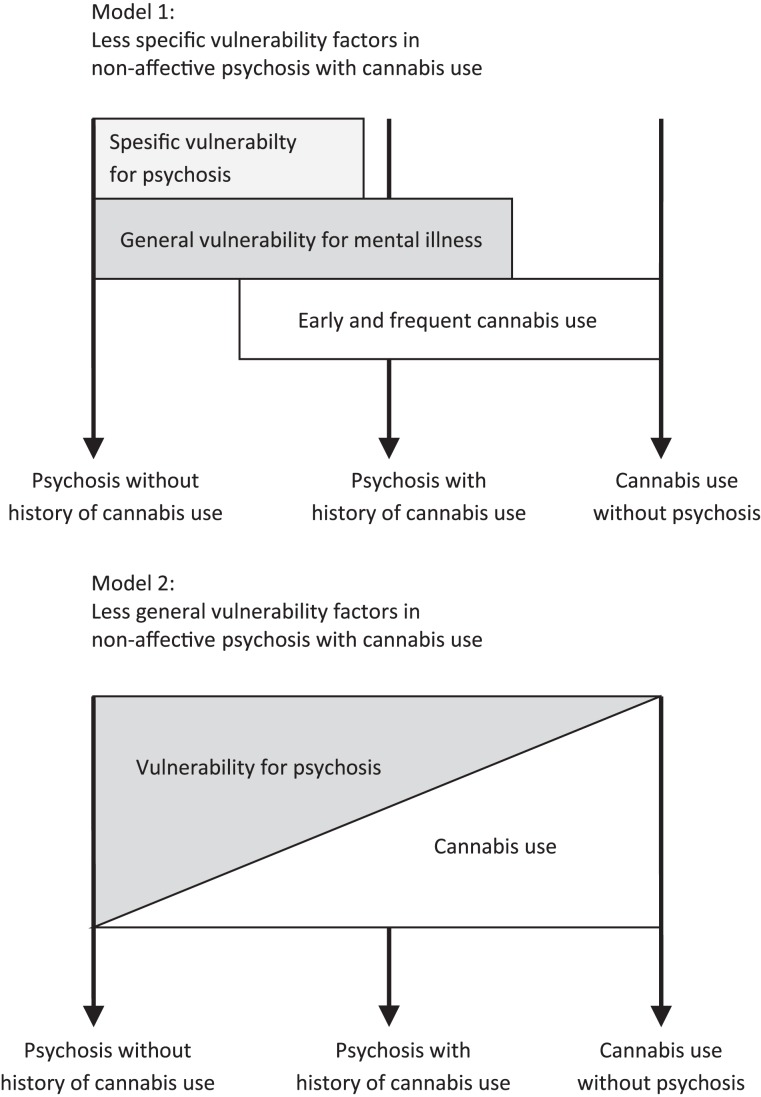
**Two alternative models for differential pathways to non-affective psychosis as a result of cannabis use and vulnerability profile**.

The first model (see model 1 in Figure 1) suggests that cannabis-using psychosis patients have an unspecific vulnerability for mental disorder in general, explaining the family loading results. There are, e.g., several findings on the unspecific family loading in mental disorders; almost any psychiatric disorder in first-degree relatives is associated with an increased risk of schizophrenia (59), and there seems to be a partly shared genetic underpinning behind several mental disorders (204). These could be related to specific domains of risk phenotypes suggested by Van Os et al. (59) such as affective dysregulation. Childhood trauma and maltreatment may also be unspecific to psychosis [see in Ref. (205)]. Cognitive dysfunction, sub-optimal brain functioning, and NSS are more specific to non-affective psychosis, and may constitute a more illness-specific vulnerability marker, seen in the more typical non-cannabis-using psychotic patients.

Still, a breakdown of information processing is central to the development of psychosis (87). Cannabis use of sufficient magnitude, or in individuals particularly vulnerable to the effects of cannabis, may lead to transient compromised brain functioning, causing a breakdown of reality testing (135, 136). These changes can cause psychosis for some individuals, but will normally not cause the characteristic persistent cognitive impairments seen in psychosis. Thus, cannabis is viewed as an environmental factor imitating the effect of the typical neurobiological vulnerability (54). In line with this, cognitive improvement was shown in those who stopped using cannabis (206).

An alternative model (see model 2 in Figure 1) could be illustrated by using the classical stress–vulnerability framework (207, 208); the tendency to develop psychosis is a function of vulnerability × stress. When cannabis is entered into the equation as a stress factor, the need for a high vulnerability load is decreased. More cannabis x less vulnerability generates a tendency to develop psychosis that is similar to less cannabis × high vulnerability. The less vulnerability load is primarily of neurobiological origin and is shown by fewer lasting cognitive deficits, fewer brain abnormalities, and fewer NSS.

## Concluding Remarks

Cannabis is a risk factor for non-affective psychosis, interacting with genetic and environmental vulnerability. Patients with cannabis use have fewer cognitive deficits, probably fewer brain abnormalities, and fewer NSS, but at the same time genetic loading and family history of mental illness comparable to other patients with non-affective psychosis. It is suggested that the pathway to psychosis via cannabis is less influenced by neurobiological vulnerability factors. Better knowledge of vulnerability profiles in those sensitive to cannabis could help us detect individuals for whom cannabis is more precarious. At this point, it can be speculated that young people with family loading of mental illness, and sub-threshold positive symptoms indicating a more general psychosis risk, should be careful with cannabis – the last point is especially relevant for early intervention services. Thus, the presence of such markers may suggest to the clinician that these patients may be particularly sensitive to cannabis. At the same time, the patients that develop psychosis after a period of cannabis use may have less neurobiological vulnerability. They may to a larger extent be able to recover their cognitive abilities if they are abstinent from illicit substances, with positive implications for the rehabilitation process and work capacity. In addition, it can also be argued that a general public warning about the age effect of cannabis use and the effect of high THC content could be put forward, but existing research does not directly test this.

## Conflict of Interest Statement

Else-Marie Løberg has received honoria in relation to the development of the Norwegian version of the RBANS by Pearson Assessment. Jan øystein Berle has consulted for Eli Lilly & Co. Norway and received honoraria from BioPhausia AB/Medivir AB, Eli Lilly & Co., H. Lundbeck AS, and Otsuka Pharmaceutical Europe Ltd. Erik Johnsen and Rune A. Kroken declare no conflicts of interests in the last 3 years. All other authors declare that they have no conflicts of interest.
